# Producing high quality cranial SRS plans with 4Pi planning technique in a commercial clinical solution

**DOI:** 10.1002/acm2.70115

**Published:** 2025-06-12

**Authors:** Ganesh Narayanasamy, Nishan Shrestha, Milan Bimali, Fen Xia, Zhong Su

**Affiliations:** ^1^ Department of Radiation Oncology University of Arkansas for Medical Sciences Little Rock Arkansas USA; ^2^ Department of BioStatistics University of Arkansas for Medical Sciences Little Rock Arkansas USA

**Keywords:** 4Pi, 4Pi SRS, acoustic neuroma, brainlab, elements, elements cranial SRS, elements image fusion, SRS, trajectory optimization, vestibular schwannoma

## Abstract

**Purpose:**

To generate high‐quality stereotactic radiosurgery (SRS) plans for single cranial lesions using 4Pi planning technique and compare these to our clinical “status quo” plans.

**Methods:**

Eighteen vestibular schwannoma (VS) patients previously planned with Varian Eclipse RapidArc and treated on a Varian TrueBeam using 6FFF MV photon beams were randomly selected. This cohort was replanned in Brainlab Elements Cranial SRS using an automatic 4Pi trajectory optimization technique (“Elements 4Pi”). “Elements ArcMatch” plans were also created, which utilized the identical arc geometry as the clinical plans in Eclipse, that is, used identical table angles, gantry start and stop angles, and collimator angles to isolate the inherent differences between the two treatment planning systems. SRS plan evaluation metrics included the Inverse Paddick conformity index (IPCI), gradient index (GI), max doses to the brainstem and ipsilateral cochlea, and number of monitor units (MUs). Pairwise comparisons between Eclipse and Elements 4Pi plans were performed using Wilcoxon signed rank test. For three‐way comparisons with Elements‐ArcMatch plans, the difference in distribution of SRS metrics was assessed first based on Friedman's test, followed by pairwise comparison if the findings from Friedman's test met statistical significance. A two‐sided *p*‐value of 0.05 was used to determine statistical significance.

**Results:**

While both Elements 4Pi and Elements ArcMatch plans had significantly lower GI, MU, and max doses to the brainstem and ipsilateral cochlea compared to Eclipse plans (*p*‐values < 0.05), Elements 4Pi also had significantly lower IPCI values.

**Conclusion:**

The automation in Brainlab Elements Cranial SRS outperformed manual expert planning and produced collision‐free and clinically deliverable plans for single targets with significantly better dose conformality, dose gradient, and lower dose to normal organs while using lower MUs compared to clinically delivered Eclipse plans.

## 1 INTRODUCTION

Intracranial malignant or benign tumors are abnormal tissue growths in the brain and can be classified as primary or metastatic. Gliomas are prevalent among malignant brain tumors and arise from the glial cells in brain. Amongst the benign brain tumors, vestibular schwannomas (VS) are not uncommon in adults and account for 5%–8% of all intracranial tumors. Also referred to as acoustic neuromas, these tumors arise from displacement of the eighth cranial nerve that extends from brain to the ear. Though benign, they can exert pressure on nerves and displace the brainstem due to “mass effect” and may also lead to significant hearing loss among other complications. Surgical gross total resection (GTR) via craniotomy may not be possible without significant neurologic morbidity.

Stereotactic radiosurgery (SRS) alone or as an adjuvant treatment to planned subtotal resections (STR), also known as adaptive hybrid surgery (AHS), has emerged as treatment options.^1^, ^2^, ^3^ SRS has shown excellent tumor control rate >91% and successfully improved cranial nerve V and VII preservation to >95%.^4^ While respecting normal tissue dose constraints, SRS allows for ablative radiation doses in a single treatment fraction by utilizing multiple coplanar and non‐coplanar beam trajectories. SRS requires very precise localization of the target (often image‐based) and can be associated with significant risk of toxicity for targets located near critical structures or in the case of large tumors.^5^ The optimal placement of non‐coplanar photon beams or arcs can be a challenging task even for experienced treatment planners. Optimizing the treatment geometry in an automated manner, mainly in the stereotactic space, is an active field of research.

4Pi, also known as trajectory‐based treatment planning, can be automated in terms of selecting the optimum treatment geometry (treatment table angles, gantry rotation spans, and collimator angles) while at the same time minimizing the risk of collision. It is based on multiple treatment priorities that need careful consideration, as some could counteract and nullify the effect of others. It maximizes target dose conformality by selecting beams with the least radiological depth to the target volume.^6^ By providing higher weightage of dosage via proximal beams, maximum target dose and dose conformality can be higher.^7^ Another important feature is that by minimizing overlap of target with specified organ at risk (OARs) along the beam's eye view (BEV), OAR dose and toxicity can be minimized.^8^ 4Pi planning has been shown to lower dose spillage volumes, as well as maximum and mean doses to proximal OARs, while meeting the prescribed objectives.^9^

The aim of this study is to evaluate the clinical adaptation and deployment of the 4Pi automated arc trajectory optimization approach of Elements Cranial SRS (Brainlab AG, Munich, Germany), which is an indication‐specific treatment planning system. It will be tested on a cohort of patients with VS that are adjacent to the brainstem, requiring careful treatment geometry considerations.

## 2 METHODS AND MATERIALS

### 2.1 Patients

Eighteen (*n* = 18) patients with VS who received SRS treatment in our clinic using Eclipse RapidArc (Varian Medical Systems, a Siemens Healthineers company, Palo Alto, CA, USA) plans on a Varian TrueBeam STx were identified in this cohort. With 9 cases positioned on either side of the brainstem, the mean ± standard deviation of volumes of the target was 1.1 ± 0.3 cc.

### 2.2 Simulation

CT simulation images were acquired in supine positioning using Brainlab stereotactic frameless masks and half Vac‐Lok (CQ Medical, Orange City, IA, USA) with arms on the side in a Philips Brilliance BigBore CT scanner (Philips Medical Systems, Andover, MA, USA), and reconstructed at 1 mm slice spacing. Gadolinium contrast‐enhanced MR imaging was performed, and T1 and T2 weighted series were co‐registered with CT simulation images in Brainlab Elements Image Fusion and exported to Eclipse version 16.1 for clinical plan generation. These scans were re‐exported along with their structure sets to Brainlab Elements Cranial SRS version 4.0.2 for plan generation. The software automatically generates an outer contour, and the planner can select objects such as immobilization mask systems and tabletop models to be added to the tissue model.

### 2.3 Treatment planning

All clinical Eclipse plans used RapidArc and involved manual placement of multiple coplanar and non‐coplanar partial arcs. Acuros XB dose calculation algorithm was used at 1.25 mm dose calculation grid spacing for treatment using 120 HD‐MLCs (high‐definition multileaf collimators) and 6FFF MV photon beams. Prescription dose (Rx) 12 Gy in 1 fraction was prescribed to the isocenter. The target objectives were 95% prescription coverage of the target (D95%) with a maximum hot spot volume of 5% of the target (D5%) irradiated to a higher dose of 15 Gy. The brainstem dose constraint was set to a maximum point dose of 15 Gy and a volumetric dose of 0.5 cc–10 Gy. The same objectives were used for generating plans in Elements; one with identical arc geometry as the clinical plans (“Elements ArcMatch”) and another using 4Pi trajectory optimization (“Elements 4Pi”), both at 1.0 mm dose grid resolution. The Elements plans were generated by matching the 98% prescription coverage which had been achieved for the clinical plans, in order to use the same plan normalization.

Elements Cranial SRS has an indication specific automated volumetric modulated arc therapy (VMAT) optimization algorithm for cranial targets and has been developed to generate an optimal arc setup geometry in terms of target coverage and OAR sparing. Both the 4Pi arc setup optimization and the VMAT algorithm in Elements Cranial SRS are indication specific, for targets in the brain. This software utilizes dedicated inverse‐optimizers that are specifically tailored for the treatment of cranial targets.^10^ In the initial setup of 4Pi arc optimization, table angles of arcs with least radiological depth are identified. In this step, as well as the gantry angular span optimization, the overlap between tumor and OAR along the beam's eye view is minimized. The collimator angle is thereafter optimized to reduce the normal brain tissue dose. By effectively utilizing the full 4Pi space around the brain, it optimizes the treatment table and gantry angles for target depth and target/OAR overlap.

The table and gantry optimization in Elements Cranial SRS generates candidate table angles which are only added to the search space if they don't increase the table‐gantry collision probability. In a second step, the table angles are tested for validity in terms of linac‐specific constraints, specifically, gantry and table angle ranges as specified by the user in the linac machine profile. As a third step, gantry and table angles are optimized stochastically in terms of radiological depth and target/OAR overlap until the optimal setup is found. Thereafter, MLC optimization is performed where the optimization algorithm utilizes iteratively adapted target rings and dose objectives to achieve steep dose gradient and optimal OAR sparing.

If needed, the planner can maneuver the plan optimization by adjusting certain slider bars in the software, as outlined in Harrer et al.^11^ The “Weighting” slider ranges from “Target” to “OAR” and enables precise control of target dose coverage by the prescription dose and meeting the OAR dose constraints. At the extreme positions of the slider, the planner can either disable any strict constraint for the most important OAR or guarantee that the prescription coverage will be fulfilled. The “Normal Tissue Sparing” slider ranges from Low to High and controls the importance of sparing the normal tissue (including OARs) surrounding the PTV. The “Modulation” slider controls the amount of modulation and while the optimizer tries to reduce the amount of modulation no matter which slider position is used; this slider sets the importance. The lowest setting deactivates the dose modulation between control points. This means disabling changes in both dose rate and gantry speed between control points. The resulting treatment plans are rather like dynamic conformal arc plans which may lead to plans with less demanding quality assurance requirements. Following dose computation using Pencil Beam or Monte Carlo, the target dose conformity index, gradient index and other metrics can be evaluated.

Clinical plans based on Eclipse RapidArc were created manually and depend on the planner's preferences in terms of treatment geometry. To study the inherent differences between Eclipse and Elements plans, “Elements ArcMatch” plans were created for the latter using identical treatment geometry as the clinical plans. By disabling the 4Pi optimization, Elements‐based ArcMatch plans were manually generated using the identical table angles, gantry rotation angles, and collimator rotation angles as the Eclipse‐based clinical plans.

### 2.4 Dosimetric comparison

Dose conformality of target and dose fall off outside target were evaluated using inverse Paddick conformity index (IPCI) and gradient index (GI), respectively, as given in Equations 1 and 2. Here, TV, VRx, and V50%Rx represent the target volume, volume irradiated by 100% Rx and 50% Rx isodose lines.

(1) $$\mathrm{IPCI}=\frac{\left(\mathrm{TV}\;X\;V_{Rx}\right)}{{\left(\mathrm{TV}\cap V_{Rx}\right)}^{2}}\;$$

(2) $$\mathrm{GI}=\frac{V_{50\%Rx}}{\mathrm{TV}}\;$$

An IPCI value close to one is considered indicative of a conformal plan. While values of GI closer to 3 are considered acceptable for larger target volumes, smaller targets can have values as high as 5.

To assess plan efficiency, the number of monitor units (MUs) was recorded as a surrogate measure of the treatment time. Radiation toxicity to OARs in terms of the maximum point doses to the brainstem and ipsilateral cochlea was recorded.

### 2.5 Treatment plan deliverability

Gamma analysis between the plan and measurements was performed based on recommendations of the American Association of Physicists in Medicine (AAPM) Task Group No. 218 (TG 218).^12^ Patient‐specific quality assurance (QA) was performed using SRS MapCHECK (Sun Nuclear, Melbourne, FL, USA), which consists of a 77 × 77 mm^2^ array with 1013 diode detectors each with active area smaller than 0.5mm × 0.5 mm. The SRS MapCHECK also has an asymmetrical placement of five detectors inside a 5 mm circular field, where the 2.5 mm detector spacing can produce a high spatial resolution necessary for SRS plan QA. The horizontal plane of the array was aligned such that the center of the target matched with the high dose region, thus providing adequate region for dose comparison between the measured and the optimized plan. Gamma analysis was performed using the SNC Patient software (Sun Nuclear, Melbourne, FL, USA) for both the Eclipse and Elements 4Pi plans delivered on the Truebeam, respectively, with a 10% dose threshold using both 3%/2 mm and 2%/1 mm dose difference and distance‐to‐agreement criteria. Passing rates were estimated based on the percentage of detectors having gamma index <1.0.

### 2.6 Statistical analysis

Descriptive statistics were reported as mean ± standard deviation. For pairwise comparison between the Eclipse and Elements 4Pi plan, Wilcoxon signed rank test was utilized to test for differences in distribution between the two plans. To conduct a three‐way comparison between Eclipse, Elements ArcMatch, and Elements 4Pi plans, one must consider the non‐negative value of dosimetric measurements. The overall difference in distribution of SRS metrics across the three plans was assessed using Friedman's test, followed by pairwise comparison if the findings from Friedman's test met statistical significance. The *p*‐values for pair‐wise comparison were adjusted for multiple comparisons using Benjamini–Hochberg procedure. A two‐sided *p*‐value of 0.05 was used to determine statistical significance. The analysis was performed in R version 4.3.2.^13^

## 3 RESULTS

In this cohort of 9 left‐sided and 9 right‐sided VS cases, mean ± standard deviation of the target volume was 1.1 ± 0.3 cc. Table 1 summarizes the dosimetric comparison of the Eclipse clinical, Elements ArcMatch, and Elements 4Pi plans.

**TABLE 1:** Dosimetric comparison of Eclipse, Elements ArcMatch, and Elements 4Pi SRS plans for 18 VS patients.

	Eclipse clinical	Elements ArcMatch	Elements 4Pi	*p*‐value Overall	*p*‐value Eclipse versus ArcMatch	*p*‐value Eclipse versus 4Pi	*p*‐value 4Pi versus ArcMatch
IPCI	1.4 ± 0.2	1.4 ± 0.3	1.3 ± 0.2	0.002	0.468	<0.001	0.012
GI	4.9 ± 0.7	3.5 ± 0.3	3.5 ± 0.4	<0.001	<0.001	<0.001	0.475
MU	6777 ± 1493	3845 ± 863	4150 ± 1019	<0.001	<0.001	<0.001	0.007
Brainstem max dose (Gy)	9.0 ± 2.0	8.0 ± 2.1	7.4 ± 2.1	<0.001	<0.001	<0.001	0.663
Ipsilateral Cochlea max dose (Gy)	6.6 ± 2.6	4.4 ± 2.5	4.4 ± 1.8	<0.001	<0.001	<0.001	0.072

**FIGURE 1:**
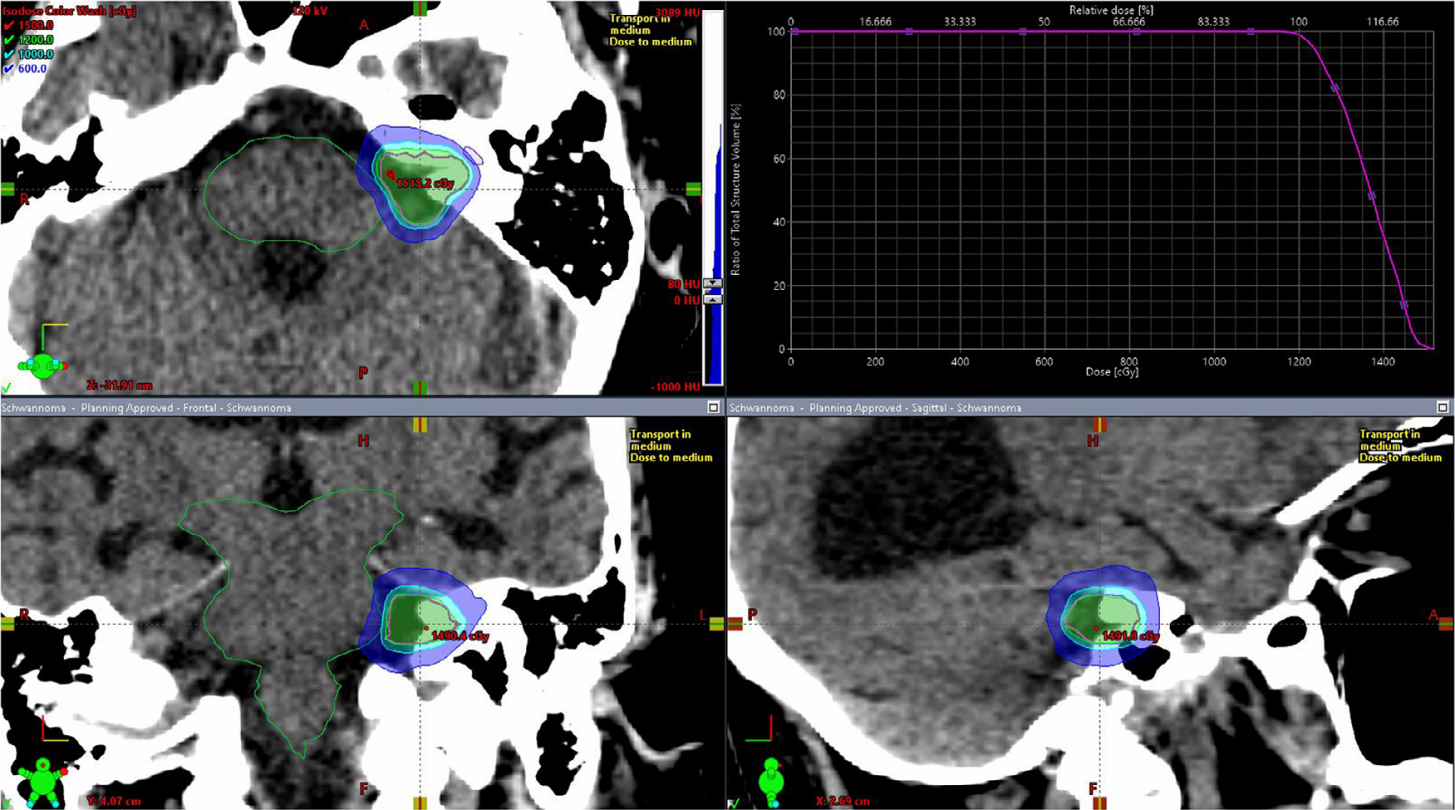
Three orthogonal views of the clinical treatment plan, along with the target DVH generated in Eclipse RapidArc Acuros XB version 16.1.

**FIGURE 2:**
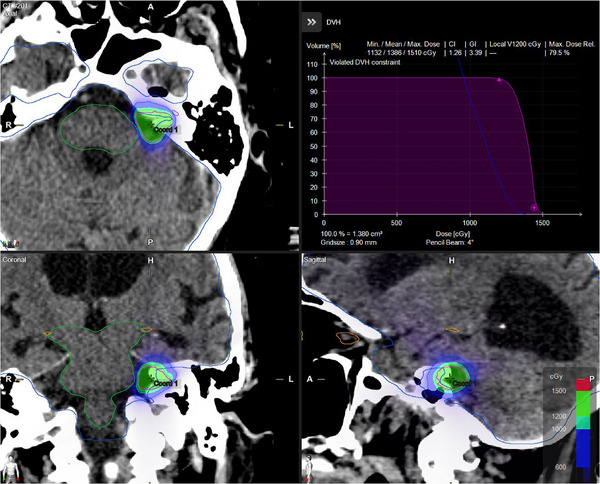
Elements “ArcMatch” plan using the same treatment geometry as the clinical Eclipse plan in Figure 1.

**FIGURE 3:**
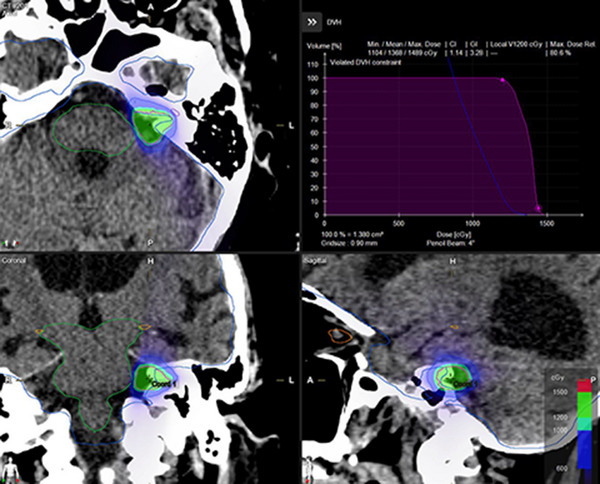
Elements 4Pi plan for the same patient is shown in Figures 1 and 2, but with automatic trajectory optimization.

**FIGURE 4:**
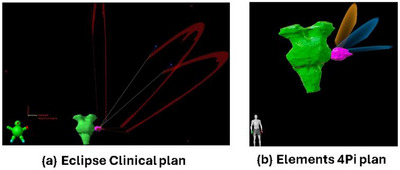
Comparison of a 3‐arc beam arrangement used in (a) Eclipse clinical plan (table angles of 30^0^, 45^0^, and 90^0^) versus (b) Elements 4Pi plan (5^0^, 40^0^, and 70^0^).

**FIGURE 5:**
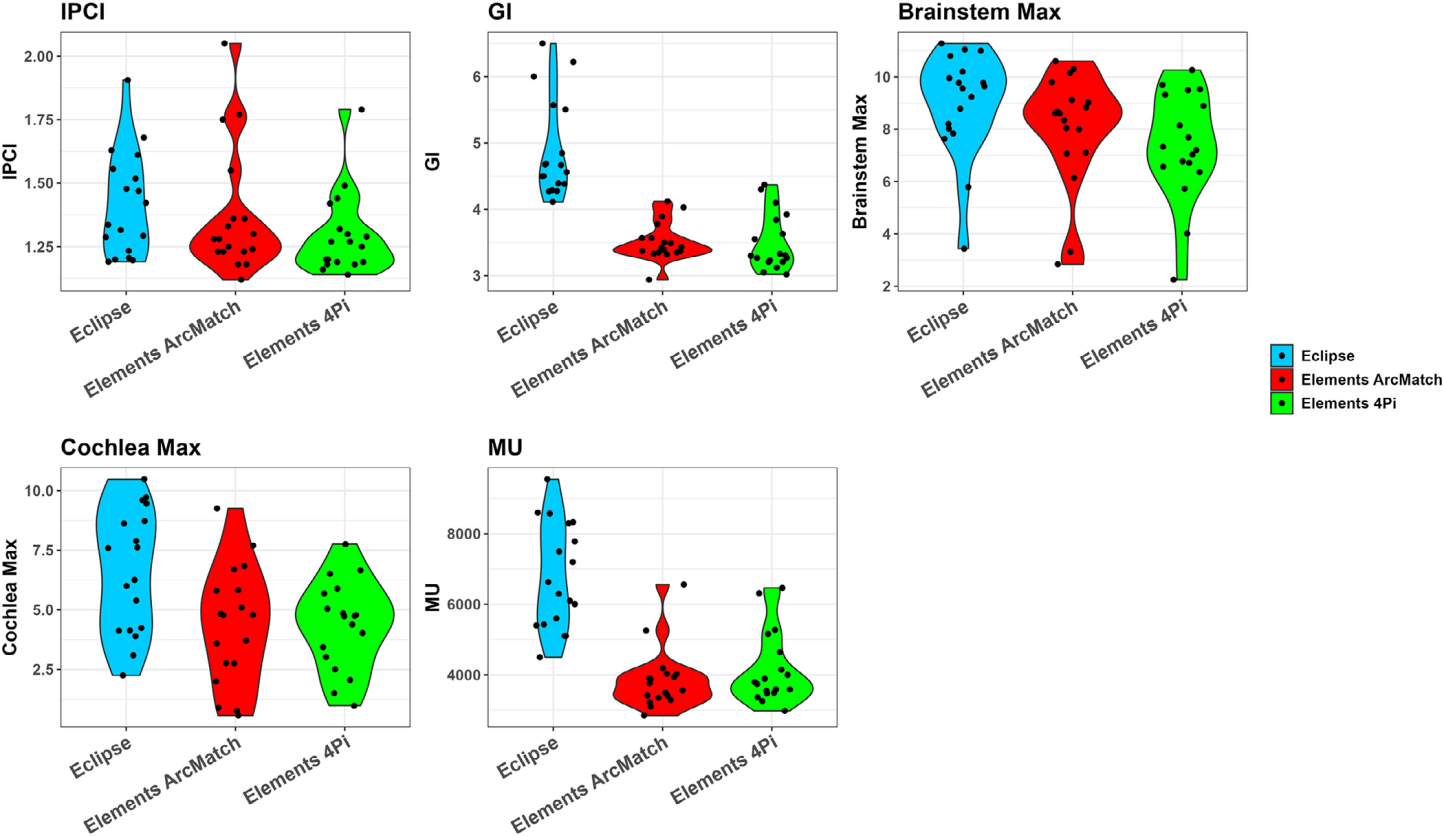
Violin plots of the inverse paddick conformity index (IPCI), gradient index (GI), number of monitor units (MU), maximum doses to the brainstem (Gy) as well as ipsilateral cochlea (Gy), for the clinical Eclipse plans (blue), Elements ArcMatch (red) and Elements 4Pi plans (green).

All Elements plans achieved the same target coverage as the clinical Eclipse plans, ensuring equivalent normalization for dosimetric comparison. Figure 1 shows an Eclipse clinical plan, while Figure 2 shows the corresponding views of an Elements ArcMatch plan, and Figure 3 of an Elements 4Pi plan for the same patient. Uniform dose distribution around the target can be observed in Figure 1, while enhanced sparing of the brainstem is noticed in Figures 2 and 3. Friedman's test returned overall *p*‐values < 0.001, confirming significant overall differences in all metrics studied. Overall, the Elements 4Pi plans had significantly better IPCI of 1.3 ± 0.2, followed by Elements ArcMatch at 1.4 ± 0.3, in comparison to Eclipse plans at 1.4 ± 0.2. With 28.3% lower GI values than the Eclipse plans (*p*‐values < 0.001), both Elements ArcMatch and Elements 4Pi achieved faster dose fall off outside the targets, resulting in lower normal tissue toxicity. In addition, the Elements ArcMatch and Elements 4Pi plans used 43.2% and 38.8%, respectively, lower MUs of the same energy (6FFF MV) photon arcs (*p*‐values < 0.001), which reduces the treatment time. Maximum point doses to the brainstem were significantly lower than the Eclipse plans by 1 Gy (11.1%) on average in Elements ArcMatch and 1.6 Gy (17.9%) in Elements 4Pi (*p*‐values < 0.001), respectively. Maximum point dose to the ipsilateral cochlea was significantly lower than the Eclipse plans by 2.3 Gy (34%) on average for both Elements ArcMatch and Elements 4Pi (*p*‐values < 0.001). Shown in Figure 4 is the comparison of beam arrangements between Eclipse clinical and Elements 4Pi for the representative patient. Figure 5 displays violin plots of the above listed metrics for the Eclipse clinical, Elements ArcMatch, and Elements 4Pi plans. All plans met the dose objectives based on QUANTEC SRS guidelines.^14^

To assess deliverability, our institutional goal of 90% passing rates uses 3%/2 mm criteria at 10% dose threshold. Using 3%/2 mm gamma criteria, all plans met the institutional acceptability criteria with passing rates > 95%. The mean ± standard deviation gamma passing rate of the Eclipse plan was 97.4 ± 1.2%, Elements ArcMatch plans at 97.8 ± 1.0%, while Elements 4Pi plans are significantly better at 98.9 ± 0.6% (*p*‐value < 0.01). It must be noted that TG 218 does not segregate IMRT/VMAT for conventional fractionation versus hypo‐fractionated SRS/SBRT plans, which require tighter tolerances.^15^ A stricter criterion of 2%/1 mm was also performed, for which average gamma passing rate of the Eclipse plans was 95.9 ± 1.9%, Elements ArcMatch 96.0 ± 1.6%, and Elements 4Pi plans 96.1 ± 1.2%.

## 4 DISCUSSION

It's a challenging task to find the optimal beam placement for a cranial target that meets dosimetric criteria for SRS and has no collision risk, even for experienced treatment planners. This dosimetric study aimed to investigate this scenario and therefore, the first step involved identifying clinical cases where the cranial tumor was in vicinity of a critical OAR. This led us to the selection of clinically delivered Eclipse SRS plans for VS, where the brainstem is a close OAR. Treatment plan generation in a dedicated and automated intracranial platform, such as Brainlab Elements, was expected to deliver equivalent if not slightly superior plans to those of manual treatment planning efforts.

The evaluated metrics of the SRS plans were derived from the native treatment planning system (TPS) that is, Eclipse plan metrics were read inside Eclipse, and Elements plan metrics inside Elements to eliminate any issues that could originate from resolution losses upon exporting the DICOM data to a third‐party software.^16^, ^,17^

One of the salient aspects of SRS treatment plan generation is in the placement of non‐coplanar arcs while providing steep dose falloff outside the target, and at the same time safeguarding the critical organs. The spatial relationship between a target and the nearest OAR can affect the dose distribution significantly. In this cohort, mean ± standard deviation of the closest distance between contours of the VS target and the brainstem was estimated to be 1.8 ± 1.5 mm. The second nearest OAR, being the optic chiasm, was estimated to be much further away at 26.7 ± 1.7 mm from the target and should therefore not have influenced the outcome of this study.

The Elements Cranial SRS software produced significantly higher quality, and clinically deliverable (as well as collision free) plans based on 4Pi trajectory optimization. In Elements Cranial SRS, the 4Pi optimization selects laterality (right versus left) based on target location such that radiological depth is minimized when irradiating the target. The number of arcs needed may vary and, in our clinic, almost all patients require a minimum of 3 arcs up to a maximum of 7 arcs, with 5 being the most used number of arcs. The delivery time depends on several factors including the total number of arcs and number of arcs with unique table angles. The Elements Cranial SRS 4Pi plans did not increase the number of arcs nor unique table angles compared to the clinical Eclipse plans and therefore, the number of MUs were recorded as a surrogate measure of the delivery time.

ArcMatch plans were also created in Elements Cranial SRS, which utilized the identical arc geometry as the clinical plans in Eclipse, that is, used identical table angles, gantry start and stop angles and collimator angles, in order isolate and study inherent differences between the two treatment planning systems.

We did not record the planning time as the clinical Eclipse plans had already been created. The time taken for a treatment plan to optimize depends on a multitude of factors, including computer specifications, software versions, as well as grid calculation size and statistical uncertainty requested of the Monte Carlo algorithm, where the latter two are both user‐defined. Others have reported on this, such as Brun et al., who stated that the computing time to generate the dosimetry is almost instantaneous for Elements Cranial SRS, but it can take several dozen minutes for VMAT in Eclipse (using the AAA algorithm for Eclipse version 13.5).^18^

The Elements 4Pi plans were of higher conformality and steeper dose gradient while utilizing lower MUs, but critical structures also showed significant dose savings. Not only were these due to treatment geometry improvements via 4Pi trajectory optimization strategy but plans with identical treatment geometry have also demonstrated substantial improvements. The significant improvements achieved in Elements ArcMatch plans in all metrics, excluding IPCI, demonstrated the superiority of the dose optimization and dose computation engine employed in Elements using identical patient treatment geometry.

In terms of the lower MU noted for the Elements ArcMatch and Elements 4Pi plans, both treatment times and modulation complexity were lowered. Treatment time savings typically leads to better patient comfort and can contribute to reduced patient motion during treatment delivery, which is welcomed especially in a single‐fraction radiosurgery setting. The VMAT technique delivers photon arcs by simultaneous modulation of gantry speed, dose rate, as well as MLC position. Highly modulated dose distributions require the MLCs to move at high speeds during gantry rotation, which often results in leaf position errors due to interleaf friction or issues in MLC motor.^19^ The elements plans have lower modulation complexity, which was also demonstrated by higher passing rates in the gamma analysis, leading to improved treatment deliverability.

The limitations of this study include the small cohort and focus on a single benign intracranial indication. Not all patients can be expected to benefit to the same extent from 4Pi optimization. This study is not powered to evaluate the impact of tumor size, tumor location, and other tumor features. While the overall results are convincing, a systematic study to investigate features that potentially influence the various beneficial aspects of 4Pi optimization will need a larger cohort with variations in tumor size and location. Another aspect of this study is that the 12 Gy isodose volume (V12), which is a primary toxicity predictor of radionecrosis, did not overlap with brain parenchyma and was not significant enough to compare between the plans.^20^ A larger study would be needed to show any potential normal brain tissue dose sparing using 4Pi optimization. Sparing normal brain tissue may lead to better retained neuro‐cognitive functioning and lower complications. Brain tumor patients with longer life expectancy, including pediatric and young adult patients, would likely benefit the most from this. Further research is needed to establish a direct correlation between dosimetric parameters and neurocognitive functional status.

## 5 CONCLUSIONS

The automation in Elements Cranial SRS outperformed manual expert planning and had significantly higher throughput that produced collision‐free and clinically deliverable plans for single targets with significantly better dose conformality, dose gradient, and lower dose to normal organs—all while using lower MUs compared to clinically delivered Eclipse plans.

## AUTHOR CONTRIBUTIONS

**Ganesh Narayanasamy**: Conception of study & proof‐reading manuscript. **Nishan Shrestha**: Writing manuscript. **Milan Bimali**: Statistics, writing manuscript. **Fen Xia**: Proof‐reading manuscript. **Zhong Su**: Proof‐reading manuscript.

## CONFLICT OF INTEREST STATEMENT

P.I. received part funding from Brainlab AG Germany.
